# Gene expression clines reveal local adaptation and associated trade-offs at a continental scale

**DOI:** 10.1038/srep32975

**Published:** 2016-09-07

**Authors:** Damiano Porcelli, Anja M. Westram, Marta Pascual, Kevin J. Gaston, Roger K. Butlin, Rhonda R. Snook

**Affiliations:** 1 Department of Animal and Plant Sciences, University of Sheffield, Sheffield S10 2TN, UK; 2 Departament de Genètica, Microbiologia I Estabdistica and IrBio, Universitat de Barcelona, Barcelona 08028, ES; 3 Environment and Sustainability Institute, University of Exeter, Penryn, Cornwall TR10 9FE, UK

## Abstract

Local adaptation, where fitness in one environment comes at a cost in another, should lead to spatial variation in trade-offs between life history traits and may be critical for population persistence. Recent studies have sought genomic signals of local adaptation, but often have been limited to laboratory populations representing two environmentally different locations of a species’ distribution. We measured gene expression, as a proxy for fitness, in males of *Drosophila subobscura*, occupying a 20° latitudinal and 11 °C thermal range. Uniquely, we sampled six populations and studied both common garden and semi-natural responses to identify signals of local adaptation. We found contrasting patterns of investment: transcripts with expression positively correlated to latitude were enriched for metabolic processes, expressed across all tissues whereas negatively correlated transcripts were enriched for reproductive processes, expressed primarily in testes. When using only the end populations, to compare our results to previous studies, we found that locally adaptive patterns were obscured. While phenotypic trade-offs between metabolic and reproductive functions across widespread species are well-known, our results identify underlying genetic and tissue responses at a continental scale that may be responsible for this. This may contribute to understanding population persistence under environmental change.

In the face of environmental change, it is critical to understand the genetic basis of local adaptation in widespread species, and the trade-offs it predicts, because remaining adapted underpins population persistence^1^. Of special concern is change in thermal environmental gradients, particularly for ectotherms in which temperature limits physiological performance^2^. In turn, thermal performance limits impact the evolution of life history traits and so fitness. Clinal gradients in phenotypes and genotypes are considered hallmarks of local adaptation and there is a rich literature documenting phenotypic trade-offs between somatic function and reproduction within populations and across clines^2^,^3^,^4^,^5^,^6^,^7^. However, despite decades of research devoted to the fundamental understanding of thermal adaptation^2^ and mechanisms of life history evolution^3^, including studying phenotypic trade-offs, their genetic, cellular and tissue connections in widespread ectotherm species remain mostly unresolved.

To begin to address this knowledge gap, large scale genomic studies have been performed to identify genetic signatures of local adaptation, including ectotherm insect models^1^ and for ectotherm insects, particularly Drosophila^8^,^9^,^10^,^11^,^12^. Gene expression studies also have been used to explore the molecular mechanisms that may underlie life history trade-offs^13^,^14^,^15^,^16^ because adaptive variation in such quantitative traits may be due to regulatory changes^17^,^18^,^19^. As gene expression is costly^20^, variation in patterns of gene expression resulting from local adaptation to a thermal gradient may reveal the modifications in resource partitioning involved in adaptive variation in life-history trade-offs^21^. With respect to genomic and global gene expression signatures to thermal adaptation, one emergent pattern is that regulation of genes involved in metabolic function alters in response to the thermal environment^22^. Given finite resources, this predicts that other classes of genes should show opposite expression patterns, reflecting life history trade-offs^14^.

Testing this prediction requires studying gene expression across a thermal gradient in many populations. However, despite the pressing need to understand the mechanistic basis of local thermal adaptation^1^,^23^, most previous studies have been limited to one or two populations and performed under controlled laboratory conditions^22^. Assessing only two populations does not permit testing the extent of adaptation to environmental gradients, as opposed to variation among populations across the species’ range for other reasons, including non-adaptive effects (although parallel responses across different continents and different species certainly help to mitigate this limitation^24^). Moreover, using populations that have long been adapted to the laboratory, and under highly controlled laboratory conditions, fails to mimic thermal selection in the wild^25^. Here we test for the trade-offs in gene expression predicted to underlie local adaptation in both common garden laboratory conditions and caged field populations, using *Drosophila subobscura* from six locations, from Valencia Spain, to Uppsala Sweden (see Supplementary Table 1) across its native 20° latitudinal range in Europe, representing an 11 °C difference in mean annual temperature. This species has been described as a “microevolutionary weapon” against climate change^26^ since it exhibits clinal variation – a hallmark of local adaptation - in both phenotypic (such as body size; Bergmann’s rule^27^) and genetic (polymorphic chromosomal inversions^26^,^28^) traits, replicated across three continents, and in genetic responses to thermal selection both in the laboratory and in the wild^29^.

## Results

We quantified gene expression from triplicated caged populations situated in the field (*in situ*) at each of six sites along the latitudinal cline, using locally-collected flies (Supplementary Figure 1a,b; Supplementary Table 1). Triplicated caged populations were also established in a common garden laboratory environment at the University of Sheffield (Supplementary Figure 1c). We collected males at a single non-stressful temperature^29^ in order to focus on thermal adaptation rather than acclimation to thermal stress. *In situ* populations were remotely monitored for temperature and males were collected, in each of two years, when the local temperature had reached 18 °C at 12 pm for three consecutive days for the first time in either the spring or autumn season. Males from replicated caged populations representing the six locations, housed continuously at 18 °C under a 12:12 light:dark photoperiod, were sampled after three generations in laboratory culture. For each sample (18 common garden samples - 6 populations, 3 cages each and 72 *in situ* samples, 6 populations, 3 cages each, sampled four times), we produced high coverage transcriptomic data (RNA-seq) on whole bodies from a pool of 20 males, which we mapped to an independent *de novo* transcriptome assembly and analysed for differential gene expression. The use of whole bodies is to provide an overall assessment of gene expression without *a priori* limitation to a specific tissue.

**Figure 1:**
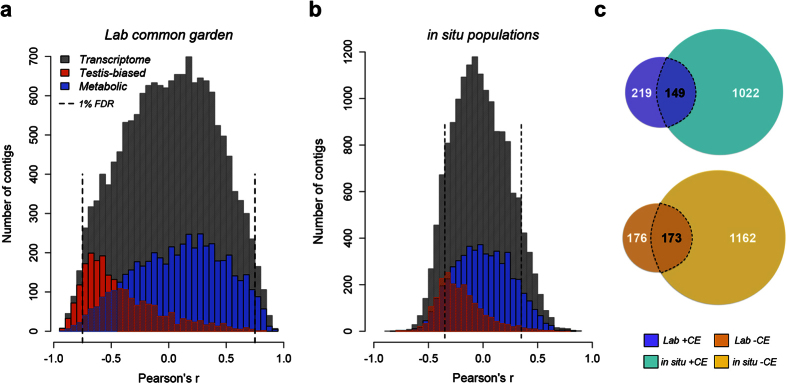
Clinal expression in *Drosophila subobscura* populations along the European latitudinal transect. (**a,b**) Distributions of correlation coefficients (Pearson’s r) between RNA-seq normalised reads and latitude for all the transcripts in the *de novo* transcriptome assembly (grey bars), putative testis-biased (red bars) and metabolic (blue bars) transcripts in six *D. subobscura* populations reared in two non-thermally stressful environmental conditions: lab common garden (**a**) and outdoor *in situ* (**b**). Positive r values denote transcripts with higher expression at higher latitudes, negative r values denote genes with higher expression at lower latitudes. The lab common garden and *in situ* datasets consist respectively of 15779 and 15947 total transcripts (grey bars), of which 1714 and 1680 are putative testis-biased (red bars) and 5257 and 5323 are metabolic homologs (blue bars). See Methods for how *metabolic* and *testis-biased* transcripts were classified. (**c**) Overlap between transcripts showing significant clinal expression (1%FDR, dashed lines in a,b) in the lab and *in situ* conditions; +CE = positive clinal expression, −CE = negative clinal expression.

We first tested the hypothesis that patterns in gene expression reflect local adaptation to long-term thermal patterns. Summarising gene expression using PCA, we found that major components of variation in both the common garden and *in situ* datasets were strongly correlated with variation in long-term thermal conditions (Supplementary Figures 2 and 3). Clinal trait variation is a strong indicator of local adaptation, so we tested for clinal variation in gene expression (CE) per transcript using Pearson correlation analysis with a false discovery rate (FDR) of 1%. Positive/negative CE (+CE/−CE) indicates expression that significantly increases/decreases with latitude. Overall, we identified 700 transcripts with CE from the common garden experiment (+CE = 368; −CE = 349; Supplementary Data 1; Fig. 1a, grey bars) and 2500 CE transcripts from the *in situ* dataset (+CE = 1171; −CE = 1335; Supplementary Data 1; Fig. 1b, grey bars).

Are the expression patterns observed in common garden conditions also seen *in situ* despite variation in thermal history and other environmental factors? If so, transcripts with clinal patterns detected in both datasets are robust candidates for a role in local adaptation (albeit this conservative approach may miss adaptive responses in the field, given the larger number of clinally expressed genes in that dataset; Fig. 1). Considering only those transcripts that responded significantly, and in the same direction, in both the *in situ* and common garden datasets, overlap of CE transcripts was much greater than expected by chance (+CE overlap: *Χ*^2^ = 595.3, d.f = 1, P < 2.2^−16^; −CE overlap: *Χ*^2^ = 612.7, d.f = 1, P < 2.2 × 10^−16^; Fig. 1c; Supplementary Data 1). We found no evidence that these clinal gene expression patterns were confounded with factors that are known to impact gene expression such as mitochondria^30^ and mtDNA haplotype^31^ (Supplementary Table 2; Supplementary Figure 4), variations in circadian rhythm genes^32^ (Supplementary Table 2), or plastic responses to thermal stress (Supplementary Table 2). Thus, we have identified a compelling set of transcripts associated with latitude, that appear to be unaffected by potentially confounding variables, and thus implicated in local adaptation to the thermal environment.

Previous work has shown that thermal adaptation results in altered metabolic investment and life history traits^2^,^23^,^33^,^34^. Here we tested whether the CE genes may indicate such trade-offs by categorising their gene ontology (GO) using FlyMine. For +CE transcripts in the common garden, 82% had annotated functions and they showed significant enrichment of biological processes related to metabolism (Supplementary Data 2). For +CE transcripts detected *in situ*, 75% were annotated and, as with the common garden, we found significant enrichment for a variety of metabolic processes (Supplementary Data 2). Given these indications of up-regulation of metabolism, we plotted the distribution of *D. subobscura* homologous metabolic transcripts arising in our common garden (Fig. 1a, blue bars) and *in situ* (Fig. 1b, blue bars) datasets. Metabolic transcripts are over-represented among +CE transcripts (common garden: *Χ*^2^ = 48.49, d.f = 1, P = 3.3^−12^, standardised residual = 7.01; *in situ: Χ*^2^ = 15.9471, d.f = 1, P = 6.5^−5^, standardised residual = 4.03) and generally under-represented in −CE transcripts (common garden: *Χ*^2^ = 7.1416, d.f = 1, P = 0.007, standardised residual = −2.73; *in situ: Χ*^2^ = 3.2803, d.f = 1, P = 0.07, standardised residual = −1.81). These results support the interpretation of genetic investment in metabolic processes in response to thermal adaptation^2^.

Once we had identified increased metabolic investment in northern populations, we predicted that this would be complemented by increased expression of genes involved in contrasting life history traits in southern populations. For –CE transcripts in the common garden dataset, 48% had annotated functions and GO analysis returned significant enrichment in biological processes related to male gamete generation, spermatogenesis, microtubule-based movement and processes, with some macromolecule modification processes (Supplementary Data 2). These are consistent with enriched cellular component terms related to microtubules and dynein complex, which are essential components for cell division, cell expansion and morphogenesis and also key components during both the mitotic and meiotic phases of gametogenesis^35^. Among the 66% of *in situ* −CE transcripts that were annotated, we found significant enrichment in the biological processes of microtubule-based movement and protein modification and cellular component categories related to mitotic and meiotic replication (Supplementary Data 2). Given these indications of up-regulation of gametogenesis, we used *D. melanogaster* and *D. pseudoobscura* datasets of genes showing testes-biased expression^36^ and plotted the distribution of *D. subobscura* homologous transcripts present in our common garden (Fig. 1a, red bars) and *in situ* (Fig. 1b, red bars) datasets. These transcripts are over-represented among –CE transcripts (common garden: *Χ*^2^ = 1131.45, d.f = 1, P < 2.2 × 10^−16^, standardised residual = 33.72; *in situ: Χ*^2^ = 440.5, d.f = 1, P < 2.2 × 10^−16^, standardised residual = 21.03) and under-represented in +CE transcripts (common garden: *Χ*^2^ = 35.1, d.f = 1, P = 3.09 × 10^−9^, standardised residual = −6.01; *in situ: Χ*^2^ = 68.0, d.f = 1, P < 2.2 × 10^−16^, standardised residual = −8.30). Overall, these results suggest increased investment in reproduction in southern populations.

**Figure 2:**
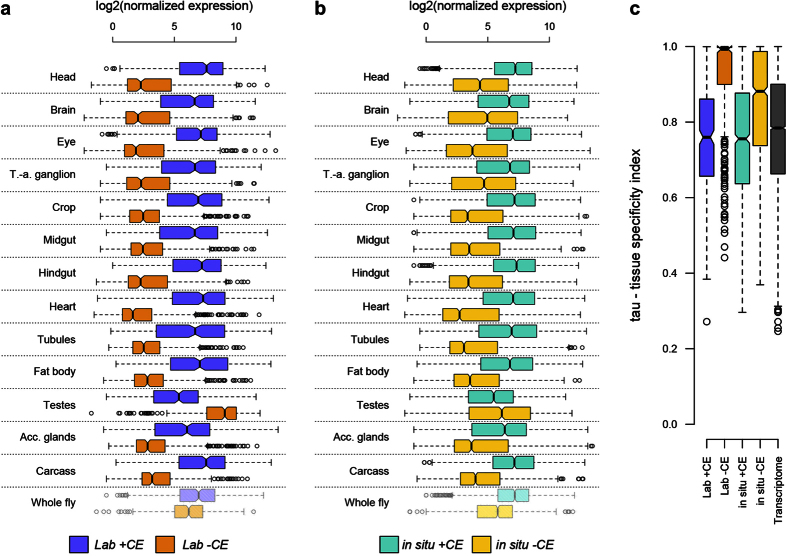
Positive and negative clinally expressed genes in *D. subobscura* have marked tissue expression differences. (**a,b**) Tissue expression profiles of significant clinally expressed genes in *D. subobscura*, estimated by using FlyAtlas^37^ expression data of *D. melanogaster* homologs. (**c**) tau (tissue specificity index) values associated with *D. melanogaster* homologs to *D. subobscura* clinally responding genes and *de novo* transcriptome assembly. tau values >0.9 are typical of genes having tissue-specific expression patterns.

Given that we found reproduction-related genes enriched among −CE transcripts, and many genes related to metabolic function in +CE transcripts, we hypothesized that −CE transcripts would show elevated testes-specific expression while +CE transcripts would be distributed across all tissues. We tested this by inferring tissue specificity associated with +CE and −CE transcripts. We interrogated FlyAtlas^37^ to identify the tissue distribution and extent of expression of *D. melanogaster* homologues of our clinally responding transcripts. Although *melanogaster* and *obscura* group flies diverged about 20 mya^38^, patterns of tissue expression for conserved, homologous genes remain highly constrained^39^, supporting the use of this cross-species comparison. We supported our hypothesis; in both datasets, expression of +CE transcripts varied little among tissues whereas −CE transcripts had lower expression in all tissues except testes (Fig. 2a,b). An index of tissue-specificity, tau, was higher in −CE than in +CE transcripts (common garden: W = 14091, P < 2.2 × 10^−16^; *in situ*: W = 383824, P < 2.2 × 10^−16^; Fig. 2c). In total, both datasets show relative increases in expression of metabolism genes in the north and reproductive genes in the south. Thus, for the first time at the gene expression level, we have identified a classic evolutionary trade-off between investment in somatic maintenance and reproduction^2^,^3^, that is adjusted by thermal adaptation across a continental scale.

Use of whole body gene expression can bias towards highly expressed genes and against genes with tissue-specific expression^40^. Nevertheless, we have revealed clear patterns that could easily have been missed in tissue-specific analyses. Allometry of constituent tissues can confound gene expression patterns^41^ but, to explain the patterns observed here, this would require negative testis allometry contrary to the positive testis allometry common in insects^42^,^43^,^44^. Moreover, our results not only mimic previous genetic and phenotypic work showing increased metabolism in the north, but also match the commonly observed range margin adaptation of increased metabolic rates that trade-off with reproduction^45^.

Because we sampled multiple sites, we can consider the shapes of clines. A linear change in expression indicates accumulated small transitions, allowing matching to a changing local optimum, whereas a stepped or sigmoid pattern suggests switching from one preferred expression phenotype to another^1^. We used nonlinear regression to describe the geographic expression clines of +CE and −CE transcripts for both the common garden and *in situ* results. We found the majority of CE transcripts exhibited a linear relationship between gene expression and latitude (Supplementary Table 3). For those transcripts showing stepped or sigmoid patterns, the sharpest environmental transition occurred in northern France, but what drives this transition is unknown. GO analysis for +CE transcripts of each cline shape found metabolism-related enrichment (Supplementary Data 3). Consistent with previous results, some enriched terms for −CE were related to male reproductive investment, particularly mating/courtship behaviour (Supplementary Data 3).

**Figure 3:**
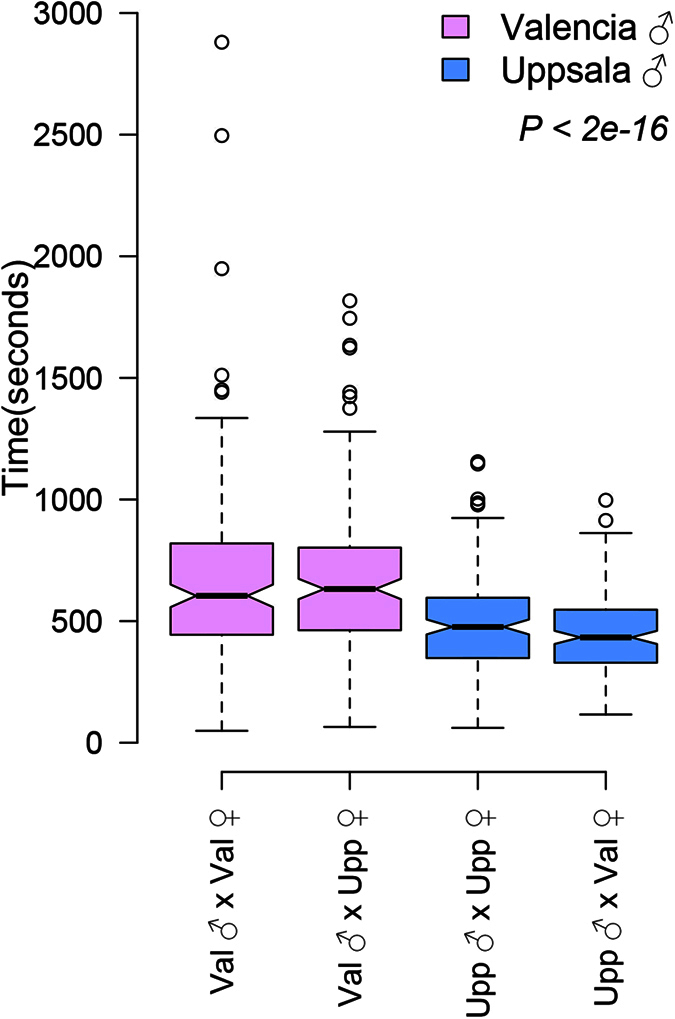
Southern *D. subobscura* males invest more in copulation than northern males. Copulation times of males from Valencia and Uppsala populations when crossed with females from the same or the alternative population.

Mating and courtship include a variety of behaviours. Females of this species are resolutely monogamous^46^,^47^, unless males fail to transfer sperm^48^, so selection arising from postcopulatory sexual selection (sperm competition and cryptic female choice) does not occur. Males need to secure mates and, when doing so, ensure sperm and ejaculate protein transfer. In Drosophila, copulation duration is heritable and typically controlled by males^49^,^50^,^51^,^52^. This trait has been used as a measure of male mating investment, primarily considered as a response to risk of sperm competition^53^,^54^,^55^ although this may not be the selective factor in monogamous species^56^. We tested the hypothesis of increased reproductive investment in southern populations in this monogamous species by measuring copulation duration for matings between Valencia and Uppsala males and females from our common garden populations. We found that Valencia males copulated for longer whether paired with either Valencia or Uppsala females (P < 2 × 10^−16^, Fig. 3). These results match previous work in this species showing that southern males mate more and for a longer time compared to northern males^57^,^58^ and that net fitness in populations experimentally evolved at higher temperatures is increased relative to those evolved at lower temperatures^29^.

Our study sampled six populations in both controlled laboratory and uncontrolled field conditions. In contrast, gene expression studies in other widespread Drosophila species tend to limit sampling to laboratory-adapted populations arising from either end of the clinal distribution. Our datasets allow us to quantify the impact of these different approaches on identifying putatively locally adaptive genes and their biological processes. We therefore re-tested our datasets using data from only Valencia and Uppsala and asked the extent to which this pair-wise comparison recapitulates the clinal analysis. Despite the significant overlap of CE transcripts between these two strategies, regardless of whether we used common garden or *in situ* data (Supplementary Figure 5, common garden: *Χ*^2^ = 2633.0, d.f. = 1, P < 2. 2 × 10^−16^; *in situ: Χ*^2^ = 6492.3d.f. = 1, P < 2.2 × 10^−16^), using data from only the ends of the clinal distribution obscures putatively locally adaptive responses. In the laboratory, using only two populations - which mimics the common experimental design – overestimates both the number of genes (Supplementary Figure 5) and the number of associated biological processes (Supplementary Data 4). From field-derived data, the use of six populations improves the ability to detect putative locally adaptive responses (Supplementary Data 4; Supplementary Figure 5). Improved detection using six populations, in either common garden or *in situ* designs, is particularly true for −CE genes in which we found clinal evidence for increased reproductive investment. In Drosophila, the number of genes that are specifically expressed in testis and are also assigned to reproductive processes is relatively small overall (~8%) so our ability to identify the gene expression trade-off between metabolic and reproductive processes may be due to finer grained resolution across the species’ distribution.

Finally, we compared our CE responses to recent microarray and RNA-seq studies of local adaptation to environmental heterogeneity in laboratory cline end populations of *D. melanogaster* and *D. simulans*^24^,^59^,^60^. We find some degree of overlap between our clinal genes and lists of differentially expressed genes identified in these studies (Supplementary Data 5). Common genes with gene annotation show a wide range of functions, although the majority of them are involved in oxidation-reduction and metabolic processes (Supplementary Data 5). Gametic investment was not identified, and only one comparison found enrichment for mating responses.

## Discussion

We found locally adaptive patterns of gene expression across the European range of *D. subobscura* with all evidence pointing to adjustment of a gene expression trade-off between reproductive and metabolic investment, which favours reproduction in the south and growth/maintenance in the north. Expression of +CE genes was high in all tissues whereas −CE genes were predominantly expressed in testes. This pattern occurred in both laboratory populations under common garden conditions and in semi-natural caged populations exposed to natural thermal variation. We also found phenotypic evidence of increased reproductive effort in southern males that were independent of the females to which they were mated. Males were examined to limit confounding variables of female oviposition status although, given the strong signals identified, we predict that females would also exhibit a metabolic-reproductive trade-off.

We used six populations to identify locally adaptive, clinal gene expression and demonstrate that our results are not impacted by known factors that alter gene expression such as mitochondrial haplotype or circadian rhythm, are not influenced by unintended stress, and are associated with long-term climatic variables, particularly temperature. The use of more than two populations facilitates describing the shape of clinal variation in gene expression across the landscape to test the extent to which populations are either matching to the local optimum, through polygenic effects, resulting in linear effects or whether selection acts on major differences in expression patterns (and is opposed by gene flow), resulting in non-linear effects^1^. The majority of clinally expressed transcripts, whether they increased or decreased expression northwards, exhibited a linear change across the landscape. Intriguingly, for those transcripts that exhibited nonlinear changes, the major transition zone was in northern France. This pattern may be an artefact of having only six populations and an increased ability to identify transitions near the middle of this distribution, although we did find nonlinear transitions at other points along the cline. Alternatively, there may be a substantial environmental selection pressure that transitions in this area. In general, only alleles under strong selection or those that are in linkage disequilibrium (LD) with those loci are expected to show such transitions^61^,^62^. In *D. subobscura*, LD may be facilitated by the large number of chromosomal inversion polymorphisms that prevent recombination when they occur as inversion heterozygotes^63^. The nonlinear transcripts identified may be in LD due to being captured in the same inversion and/or represent independent selection responses given that some chromosomal inversions show larger shifts in frequency in northern France^64^. Genomic resources for this species are not available to test for the distribution of these transcripts, although we hope that developing *D. subobscura* as a model genomic system will be a funding priority given the role this species has played in documenting replicated, real-time genetic responses to climate change^28^.

We compared our *D. subobscura* differential gene expression results to other microarray and RNA-seq studies of *D. melanogaster* and *D. simulans* in the context of local thermal adaptation. There is little overlap when comparing our RNA-seq data and *D. melanogaster* microarray data. Across the two RNA-seq studies, there is significant overlap in differentially expressed genes with both our common garden and *in situ* results. Many of these genes are not annotated, so we compared common GO terms between studies and consistently found metabolic-related functions as recurrent biological processes. The significant overlap is reassuring as we found that using only data from cline end populations overestimates putative locally adaptive transcripts in laboratory conditions and underestimates them in field conditions. However, a cautionary note is necessary. There was low overlap between studies in reproductive-related genes or GO terms and we found that clinally varying expression in reproductive processes were identified more consistently when using the 6-population compared to the 2-population datasets. This suggests that previous studies using only cline tips may have been unable to detect putative trade-offs between metabolic and reproductive gene expression.

Fitness trade-offs between survival and reproduction are well-documented^2^,^4^. We find clinal gene expression trade-offs between metabolic- and reproduction-related functions and we suggest that this provides a mechanistic link to a macroecological pattern of latitudinal variation in intrisinc population growth rate. Some studies have shown that species have higher intrinsic population growth rates at lower latitudes and one explanation, which our data support, has been described as the “warmer is better” hypothesis or the “tyranny of thermodynamics”^34^. This hypothesis states that while physiological adaptation to cold allows ectotherms to invade cold environments, they cannot compensate for reduced rates of maximal production because of energetic and thermodynamic constraints on investment in reproduction^34^. Previous work in this species found that replicate populations evolving at three different temperatures diverged in both thermal optimum and net fitness^29^. Our data may provide a mechanism to explain increased productivity in lower latitude populations, relative to higher latitude populations where the trade-off between investment in somatic maintenance and reproduction must be adjusted in favour of maintenance. Understanding the genetic constraints associated with these trade-offs may have important implications for forecasting how populations will respond to climate particularly for northward range movement^65^,^66^.

## Methods

### Species used and the establishment of wild and laboratory populations

We focus on *Drosophila subobscura* because it is a “microevolutionary weapon to monitor global change” with respect to genetic composition of populations^26^. It exhibits chromosomal inversion and phenotypic clines, replicated in its native distribution in Europe and in both North and South America following independent colonization events^67^. In Europe, chromosomal inversion frequencies have changed in response to real-time climate change, most probably in response to increasing temperature^28^. Clinal patterns of variation are often ascribed to selection, including latitudinal clines for increasing body size as latitude increases observed in this species^27^. Females of this species are monogamous^46^,^47^, although females will need to mate again if the first mate does not transfer a functional ejaculate^48^. There is no evidence of diapause^68^,^69^.

We sampled wild *Drosophila subobscura* populations from six European locations encompassing twenty latitudinal degrees (from Valencia, Spain, to Uppsala, Sweden (see Supplementary Table 1), representing the majority of the species’ western European latitudinal range^70^ by net sweeping on fermented banana baits between August and September 2011.

#### In situ

From each location, 150 females were collected and three replicated cultures (50 females/cage) were established (total of 18 cultures). Cages were wooden-framed (approximately 125000 cm^3^), surrounded by insect netting and furnished at the bottom with a layer of autoclaved sand covered with a layer of autoclaved wooden bark. Populations were reared on instant Drosophila medium (Carolina Biological Supply, Burlington, NC) in plastic bottles (250 ml), in which a total of 60 ml of final medium (1 volume of instant media: 1 volume of dH_2_O) were placed in each cage and then removed after 30 days. Every seven days, two new food bottles were placed in the cage. At each site, project partners managed this process.

For each population, the three replicated cages were housed in experimental structures shaded from direct sun and provided with an “OmniText -TDP4 GSM Logging and Alarm Unit” (Omni Instruments Ltd, UK) which monitored temperature and sent, via satellite to a dedicated webpage, current temperature data collected every 10 minutes from the cages (see Supplementary Figure 1). These data were used to monitor local conditions for determining when to sample. An HOBO data logger (Onset Computer Corporation) also was placed inside cage 1 of each population, to provide additional temperature and humidity data. These populations were sampled four times across two years (see “RNA-seq reads mapping and normalization” for final library number for analysis).

Cages were closed following each sampling period to avoid thermal selection by heat and cold stress. Re-establishment of populations occurred prior to the start of the next sampling season by transferring adult *D. subobscura* females (100/cage) from laboratory populations (see below) and transporting them back to their place of origin, to be cultured *in situ* as described above. In this way, selection for extreme temperature tolerance was eliminated and migration between populations and cages did not occur. Thus, our data represent replicated populations, across multiple seasonal samples, that are unaffected by selection and migration.

#### Common garden

After the first season of sampling, flies from each cage were brought to the University of Sheffield, and set up in a constant temperature unit of the Grodome greenhouse at the Arthur Willis Environment Centre, University of Sheffield (Supplementary Figure 1c). Populations were allowed to grow with overlapping generations (around 24 days) at 18 °C, which is considered to be the optimal rearing temperature for this species^29^, with a 12:12H light:dark cycle. Laboratory population densities were in the range of 500–2000 individuals per cage and were provided with the same food source as field flies.

### Sampling

#### In situ

To avoid thermal tolerance selection during episodes of extreme cold (winter in the north) and heat (summer in the south), *in situ* cages were closed each season and re-seeded at each site with common garden flies from the appropriate population. Populations were left to grow for five to eight weeks on instant Drosophila medium, as above, so that the subsequently sampled adults were derived from these semi-natural conditions. During the spring seasons, adult males were collected when the temperature in the cages reached 18 °C at noon across three consecutive days. Likewise, males were collected during the autumn seasons when the temperature in the cages was lower than 19 °C (optimal = 18 °C) for three consecutive days. Collections took place at noon the following day. Upon collection, flies were snap frozen on site and transported to the project partner laboratory in dry ice, stored in a −80 °C freezer, and then shipped to the University of Sheffield in dry vapor shippers (CryoPort, Inc.) for RNA extraction. Project partners were trained by DP to ensure that fly handling was standardised across the sites. Four samples were collected in each population and replicate: in autumn 2011 and 2012, and spring 2012 and 2013.

#### Common garden

After three generations in the laboratory, approx. 80 flies from each cage were collected, placed in 50 ml Corning tubes, snap frozen in liquid nitrogen for 10 seconds and immediately moved into dry ice, from where they were then transferred into a −80 °C freezer until required for RNA extraction.

### Sample preparation and RNA purification

For each collection from the common garden and *in situ* experiments, six pools of five males from each cage were placed in 2 ml Eppendorf Safe-Lock Tubes to provide RNA intended for studying differential gene expression. Sequencing costs prevented us from processing both sexes so we focused on males because they are less likely to vary in their reproductive state than females. Each sample was provided with a single 5 mm sterile RNase-free stainless steel bead (QIAGEN) and suspended in 1ml Trizol (Life Technologies). Samples were disrupted using a TissueLyser (QIAGEN) for 5 min at 30 Hz. Total RNA purification was performed according to the Trizol-manufacturer’s instructions and followed by a clean-up step using RNeasy Mini Kit (QIAGEN) supplemented with RNase-Free DNase Set (QIAGEN). Total RNAs were then checked for quality and concentration using both a 2100 Bioanalyzer (Agilent Technologies, Inc.) and a NanoDrop 8000 spectrophotometer (Thermo Fisher Scientific, Inc.). For each cage-collection, equal amounts of total RNA (2.5 μg) from the best four RNA samples were pooled together to produce a final 20-male RNA pool per cage (10 μg of total RNA). Pools were used for expression analysis to ensure sufficient high-quality RNA and to reduce variance in expression due to individual differences. Furthermore, from the common garden collection, twelve males each from the Valencia and Uppsala populations were individually placed in separate, frozen 2 ml Eppendorf Safe-Lock Tubes and RNA extracted to generate a *de novo* transcriptome assembly. RNAs from single individuals were not pooled at this stage but shipped, along with the pooled samples, to the NBAF GenePool sequencing facility in Edinburgh (Scotland) using dry vapor shippers (CryoPort, Inc.).

### cDNA library preparation, sequencing and reads QC

At the GenePool facility, two single-male RNA samples from the Uppsala and three from the Valencia population were pooled separately to produce two final RNA pools for the *de novo* transcriptome assembly, hereafter named *Uppsalapool* and *Valenciapool,* respectively. All the cDNA libraries were prepared using the TruSeq RNA Sample Prep Kit (Illumina, Inc.) and the Illumina manufacturer’s protocol for multiplex sequencing was followed. Libraries obtained from *Uppsalapool* and *Valenciapool* samples were sequenced on two HiSeq 2000 lanes of 100-bp paired-end reads resulting in ~80 M read pairs per pool. 18 and 72 libraries obtained from the 20-male RNA pools of the common garden and *in situ* experiments were sequenced on two and five HiSeq 2000 lanes of 50-bp single-end reads resulting in an average of ~17 M and ~14.5 M reads per pool, respectively. After sequencing, raw reads were filtered and trimmed of adapter sequences using the fastq-mcf tool of the ea-utils software package (https://code.google.com/p/ea-utils) with a quality cut-off of 20.

### *De novo* transcriptome assembly

Assembly was carried out using the Trinity assembler^71^. High quality paired-end reads from the *Uppsalapool* and *Valenciapool* were digitally normalised with a max coverage of 50 before initiating the assembly process. For each read pool, a first raw assembly with standard settings was blasted against the NCBI non-redundant (nr) protein database in order to detect contaminant sequences from species not related to the Drosophila genus. Contaminant contigs were retained as a separate pool of sequences, which were used to clean contaminant reads from those originally used in the *de novo* transcriptome assembly with the GSNAP tool^72^ (*--fails-as-input* option). Filtered reads were then pooled together from the two populations, digitally normalised as above and assembled into 91164 contigs using Trinity (with the *--jaccard_clip* option to reduce transcript fusion due to UTR overlap). Redundant and extremely lowly expressed contigs were removed by using, respectively, the CD-HIT tool^73^ (95% identity) and the *filter*_*fasta*_*by*_*rsem*_*values.pl* Trinity-utility (retaining sequences with Isopct ≥ 1.00 and fpkm ≥ 1.00), resulting in a pool of 57929 transcripts. Since our analyses were focused on the gene level, from each Trinity component (cluster of transcripts from the same gene or highly similar paralogs) we selected one representative among those returning identical BLAST matches against the NCBI nr protein database of Drosophilids (*blastx -num*_*alignments 3* -*evalue 1e-5*). The selection was made on the basis of the BLAST score and/or maximum length. If the BLAST returned a single match or if the alignments with multiple matches were nested one in the other(s), the selected sequence was moved directly into the final transcriptome bin. If there was partial or no overlap between the alignments with multiple matches, as in the case of fused transcripts, the putative coding sequences were extracted, pooled together in a dedicated bin and then processed with the CD-HIT tool (95% identity), before being moved into the final transcriptome bin. This process produced a final reference transcriptome consisting of 21728 contigs (N50 = 2000). We generally refer to these contigs as transcripts.

### Mitochondrial genome assembly

From our *de novo* transcriptome assembly we retrieved *D. subobscura* mitochondrial transcripts by using Blast against the *D. pseudoobscura* mitochondrial genome (*blastn -evalue 1e-50*). We then fed these transcripts to *GapFiller*^74^ (default settings) together with *Uppsalapool* and *Valenciapool* paired-end reads and generated a 14816 bp assembly showing 92% identity to the *D. pseudoobscura* mitochondrial genome. This assembly represents to the transcribed portion of the *D. subobscura* mitochondrial genome.

### RNA-seq reads mapping and normalization

We mapped high quality reads deriving from the 18 libraries obtained from the common garden (6 populations × 3 cages/population) and the 72 libraries from the *in situ* (6 populations × 3 cages/population × 4 sampling times) samples onto our *D. subobscura* reference transcriptome using the GSNAP tool. Derived SAM files from uniquely mapping reads were utilized to generate two separate tables of counts, one for the common garden and one for the *in situ* samples, which were separately processed with the Bioconductor software package edgeR^75^ in order to filter out transcripts with very low counts and then normalize the data. Using an expression cut-off of 2 counts per million reads for each transcript, from the lab common garden and *in situ* datasets we retained, respectively, 15779 and 15947 transcripts. Read counts were normalized for each dataset using the Trimmed Mean of M-values (TMM) method, implemented in edgeR, to adjust for variation in library sizes^76^.

### Functional annotation of transcripts

In order to characterize the putative function of *D. subobscura* transcripts, transcripts from the *de novo* transcriptome assembly were blasted against the NCBI nr protein databases of *D. melanogaster* and *D. pseudoobscura (blastx -evalue 1e-5*). Homologies were found with 11730 *D. melanogaster* and 11696 *D. pseudoobscura* protein coding genes and were employed for subsequent data analyses (e.g. GO term enrichment analysis).

### Statistical analyses

We analyzed the common garden and *in situ* samples separately. After normalization, the two dataframes (common garden and *in situ*) were completed by adding geographical (latitude) and climatic (yearly mean temperature, relative humidity and precipitation) variables (these latter variables were generated using climate records of last 30 years from the closest weather stations to each sampling location using the web resource http://www.tutiempo.net/en/Climate/). All the statistical analyses were performed using R version 3.1.1 77.

To examine overall gene expression and environmental variables, we first determined the correlation between latitude and yearly mean temperature (Pearson correlation test, *cor.test* function; r = 0.9989, df = 4, P = 1.728 × 10^−6^). We then used Principal Component Analysis (PCA), using the *prcomp* function, to generate PC1, PC2 and PC3 (describing approximately 40% of variability in both datasets), which were then correlated with latitude (temperature proxy) and two other climatic variables (yearly mean relative humidity and precipitation) using a Pearson correlation test.

Clinal gene expression was investigated by testing the relationship of transcript-specific expression with latitude using diverse approaches. We implemented correlation tests to determine the clinal expression trends for each transcript (if highly expressed in the North/cold climate and low in the South/warm climate, or the reverse) and to retrieve associated p-values. Tests for normality (*shapiro.test* function) of the expression level distribution showed departures in 0.6% of genes in the common garden dataset and 31.6% of genes in the *in situ* dataset at false discovery rate (FDR) of 1%. Pearson (linear) and Spearman (rank) correlations on both datasets resulted in very similar outputs: the correlation between coefficients was 0.98 for the lab common garden and 0.97 for the *in situ* datasets. Here we report results from the Pearson correlation. FDR correction of 1% was applied using the *p.adjust* function. The larger number of clinally responding genes detected *in situ* is likely to result from increased power (4 replicate time points per location: averages from single time points in the *in situ* data were +CE = 702, −CE = 590).

In order to explore the form of the relationship between gene expression and latitude, we used non-linear regression and Akaike Information Criterion (AIC) to compare the likelihoods of step, sigmoid, linear and null models. Selection on an environmental gradient may produce clines for individual loci or traits that are steeper than the rate of environmental change and maintained by a balance between selection and dispersal^1^,^78^. These clines are expected to be sigmoid in form, modelled here with a tanh curve, but will appear as a step change in expression between a pair of sites if the sigmoid curve is too steep to resolve with our sampling. Dispersal-independent clines^79^, where the gene expression level matches the local environment, are expected to be linear. We compared these models with a null model of constant gene expression by using the *mle2* function in the R package *bbmle*^80^. We first rescaled [0 to 1] the read counts for each transcript as follows


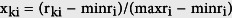


where *x*_*ki*_ is the rescaled expression value in the sample *k* for the transcript *i, r*_*ki*_ is the read count in the sample *k* for the transcript *i, minr*_*i*_ and *maxr*_*i*_ are, respectively, the lowest and highest read counts for the transcript *i*.

We obtained maximum likelihood estimates for each transcript expression level by implementing the following functions.


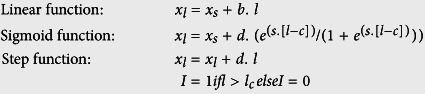


where *x*_*l*_ is the rescaled expression at latitude *l* (expressed relative to the southernmost site), *x*_*s*_ is the rescaled expression in the southernmost site, and the fitted parameters are: *b* – the linear regression coefficient, *d* - the difference in rescaled expression between the southern and northern extremes of the cline, *s* – the maximum slope, *c* – the centre, and *l*_*c*_ = the position of the step change. Maximum likelihood estimates were then converted into AIC values to select the model with the lowest AIC value, for each transcript.

Pair-wise differential gene expression between the two end populations (Uppsala and Valencia) was performed using edgeR with default settings and 1%FDR cut-off.

Both +CE and −CE are potentially signatures of local adaptation. However, rather than an adaptive response, CE could be an artefact of clinal variation in mtDNA haplotype^31^ (see below), variation in gene expression associated with the mitochondrion^30^, circadian rhythm^81^, heat or cold stress, and/or differences in seasonality across sites (although this could not explain common garden results). Functional enrichment in CE transcripts for these classes of genes was tested using the Fisher’s exact test (*fisher.test* function). This same test was used for testing the significance in gene overlapping between our study and published studies on other Drosophila species^24^,^59^,^60^.

To test for enriched tissue expression (tau^82^, see below), and for copulation duration, the Wilcoxon–Mann–Whitney test was employed (*wilcox.test* function). We used chi-square statistics (*chisq.test* function) to analyze the overlap between CE transcripts in the common garden and *in situ* datasets and to detect over- and under-representation of metabolic and testis-biased genes in CE transcripts.

### Locally adaptive GO enrichment analysis and retrieval of functional gene sets

GO terms enrichment analyses were performed using FlyMine^83^. Functional gene sets were retrieved from the Gene Ontology Consortium (http://geneontology.org), using specific GO terms or IDs and applying a taxon filter. To assess potential dependence of clinal expression on specific gene functions, rather than local adaptation, we made use of *D. melanogaster* gene sets belonging to “mitochondrion”, (GO id: GO:0005739) and “circadian rhythm” (GO id GO:0007623), and to genes associated with heat or cold stress deriving specifically from the CESAR dataset (http://pearg.com/cgdfront/), which collects genes associated with environmental stress response. Furthermore, *D. melanogaster* genes belonging to “metabolic process” (GO id: GO:0008152) were used to identify overall clinal expression of *D. subobscura* metabolic genes (transcripts) by implementing homology conversion, see blue bars in Fig. 1a,b.

### Tissue gene expression

We downloaded the full tissue gene expression dataset from FlyAtlas^37^ (http://flyatlas.org/atlas.cgi) and retrieved tissue mRNA signals of *D. melanogaster* homologues to highly significant (1%FDR) *D. subobscura* clinally expressed genes. These data were also used to calculate the graded tissue specificity index^82^, tau, for each 1%FDR transcript, and for extrapolating *D. melanogaster* testis specific genes, using an expression fold change of 50 as cut-off. We also made use of a *D. pseudoobscura* dataset for genes showing testis-biased expression^36^, retaining genes with an expression fold change cut-off of 256 (log2(8)), in order to provide a more complete annotation of putative testis-biased transcripts in *D. subobscura*, see red bars in Fig. 1a,b.

### Copulation duration

In November 2012, we established five isofemale lines each from the Valencia and Uppsala lab populations on standard Drosophila media in vials, cultured in an incubator at 18 °C with a 12:12H light:dark cycle. After three generations the lines were assessed for variation in copulation duration, which is the amount of time a male spends copulating and can be considered as an estimate of the male’s investment in the mating^84^. To measure copulation duration, virgin males and females were collected and transferred separately into new vials containing standard food medium and live yeast and left to develop for 6–7 days until they were sexually mature.

A virgin female was placed with a virgin male in a fresh food vial and the start and end of copulation were recorded. We performed a fully factorial crossing scheme in which we measured copulation duration in 9 matings per line combination (900 matings in total) and tested for significance via two-way ANOVA analysis using the *aov* function: Copulation~Male*Female).

### mtDNA SNP anaysis

Mitochondrial DNA sequence variation might have an effect on gene expression^30^. We therefore examined the frequencies of two common *D. subobscura* mtDNA haplotypes identified in earlier work, which can be distinguished using a polymorphic *HaeIII* cut site. If haplotype frequencies were similar across locations, it is unlikely that any clinal patterns in gene expression were caused by clines in mtDNA variation. We tested this hypothesis using both common garden and *in situ* RNA-seq data. Replicates were analysed separately. After removing adapter sequences, RNA-seq reads shorter than 35 bp or with an average quality less than 20 were discarded. Trimming and filtering were performed using the program ea-utils. The remaining reads were mapped onto our *D. subobscura* mitochondrial genome assembly using bwa-mem^85^ with default settings. Mapped reads with a mapping quality less than 30 and secondary hits (short alternative alignments) were discarded in samtools^86^ v. 1.2 (http://www.htslib.org/). Potential PCR duplicates were removed using PicardTools MarkDuplicates (http://broadinstitute.github.io/picard/). After that, samtools was used to combine per-sample bam files into a single mpileup file, which was then converted into the “sync” format associated with the PoPoolation2 package^87^. We identified the polymorphism in the mtDNA *HaeIII* cut site (“A” allele in our assembly) using sequence information provided in Castro *et al*.^88^ and calculated its frequency for each sample. Samples with a coverage depth <10 were discarded.

Data accessibility: Our *de novo D. subobscura* transcriptome and reads data are available at the NCBI BioProject PRJNA325922.

## Additional Information

**How to cite this article**: Porcelli, D. *et al*. Gene expression clines reveal local adaptation and associated trade-offs at a continental scale. *Sci. Rep.* **6**, 32975; doi: 10.1038/srep32975 (2016).

## Supplementary Material

Supplementary Information

Supplementary Data 1

Supplementary Data 2

Supplementary Data 3

Supplementary Data 4

Supplementary Data 5
