# Supplementary material for: Gene expression clines reveal local adaptation and associated trade-offs at a continental scale
Source: Sci Rep. 2016 Sep 7;6:32975. doi: 10.1038/srep32975 (PMC5013434; doi:10.1038/srep32975)
Supplement: Supplementary Information [file srep32975-s1.pdf]

Supplementary information to:

Gene expression clines reveal local adaptation and associated trade-offs at a continental scale

Authors:

Damiano Porcelli[1†*], Anja M. Westram[1], Marta Pascual[2], Kevin J. Gaston[3], Roger K. Butlin[1], and Rhonda R. Snook[1*]

Affiliations:

[1]Department of Animal and Plant Sciences, University of Sheffield, Sheffield S10 2TN, UK

[2]Departament de Genètica, Microbiologia I Estabdistica and IrBio, Universitat de Barcelona, Barcelona 08028, ES

[3]Environment and Sustainability Institute, University of Exeter, Penryn, Cornwall TR10 9FE, UK

*Correspondence to: D.P. (dp519@cam.ac.uk; damiano.porcelli@gmail.com); R.R.S. (r.snook@sheffield.ac.uk)

[†]Current address: Department of Physiology, Development and Neuroscience, University of Cambridge, Cambridge CB2 3DY, UK

Supplementary Figures:

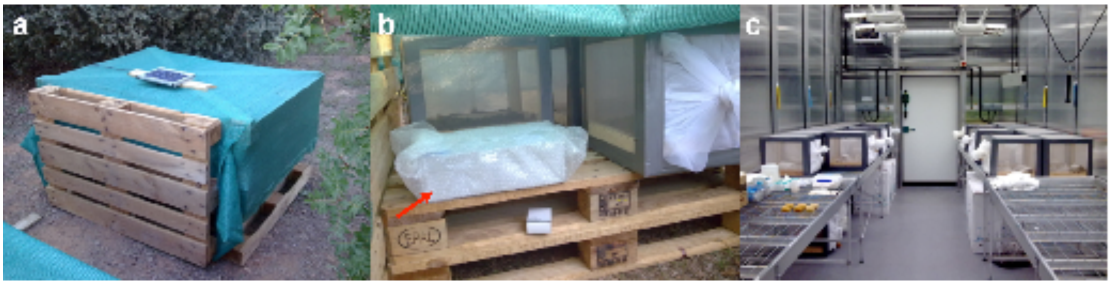

Supplementary Figure 1. **Lab common garden and *in situ* experimental settings**. (a,b) in situ populations were kept in wooden shaded structures built by means of pallets. For each location and population, we accommodated three replicated fly cages in these constructions also equipped with an OmniText -TDP4 GSM Logging & Alarm Unit (red arrow) in order to collect live temperature data from inside each cage. (c) In Sheffield, we set up a common garden experiment where all the populations (triplicated) were kept in the same thermal condition (see Methods).

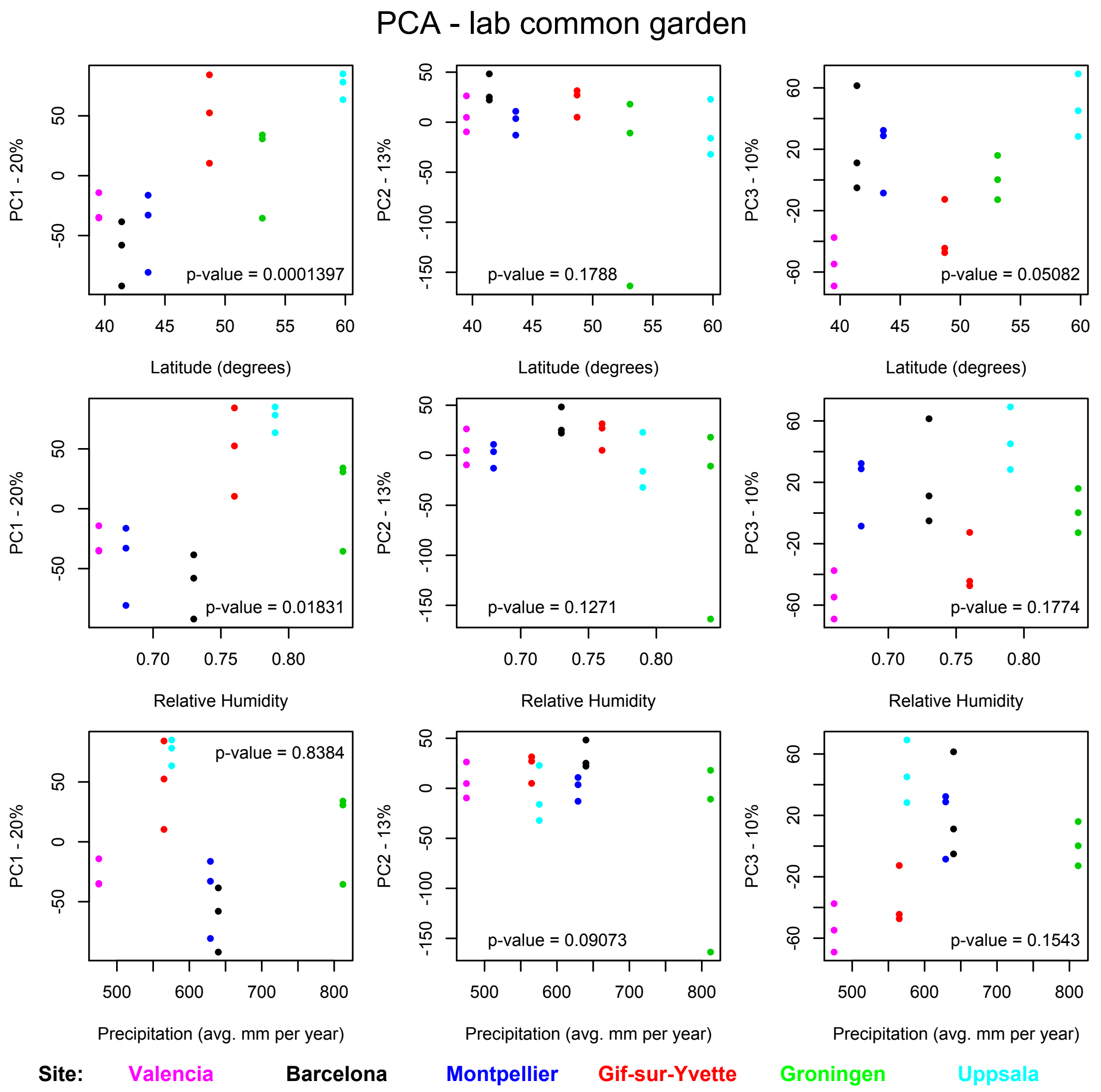

Supplementary Figure 2. **Association between principal components (PC) of lab common garden *RNA-seq* data and abiotic variables.** The three principal components from the PCA analysis on lab common garden RNA-seq data were modelled with latitude, which also serves as a proxy for yearly mean temperature (these two have a Pearson's r of -0.9989, see first row of plots), yearly mean relative humidity (second row of plots) and precipitation (third row of plots).

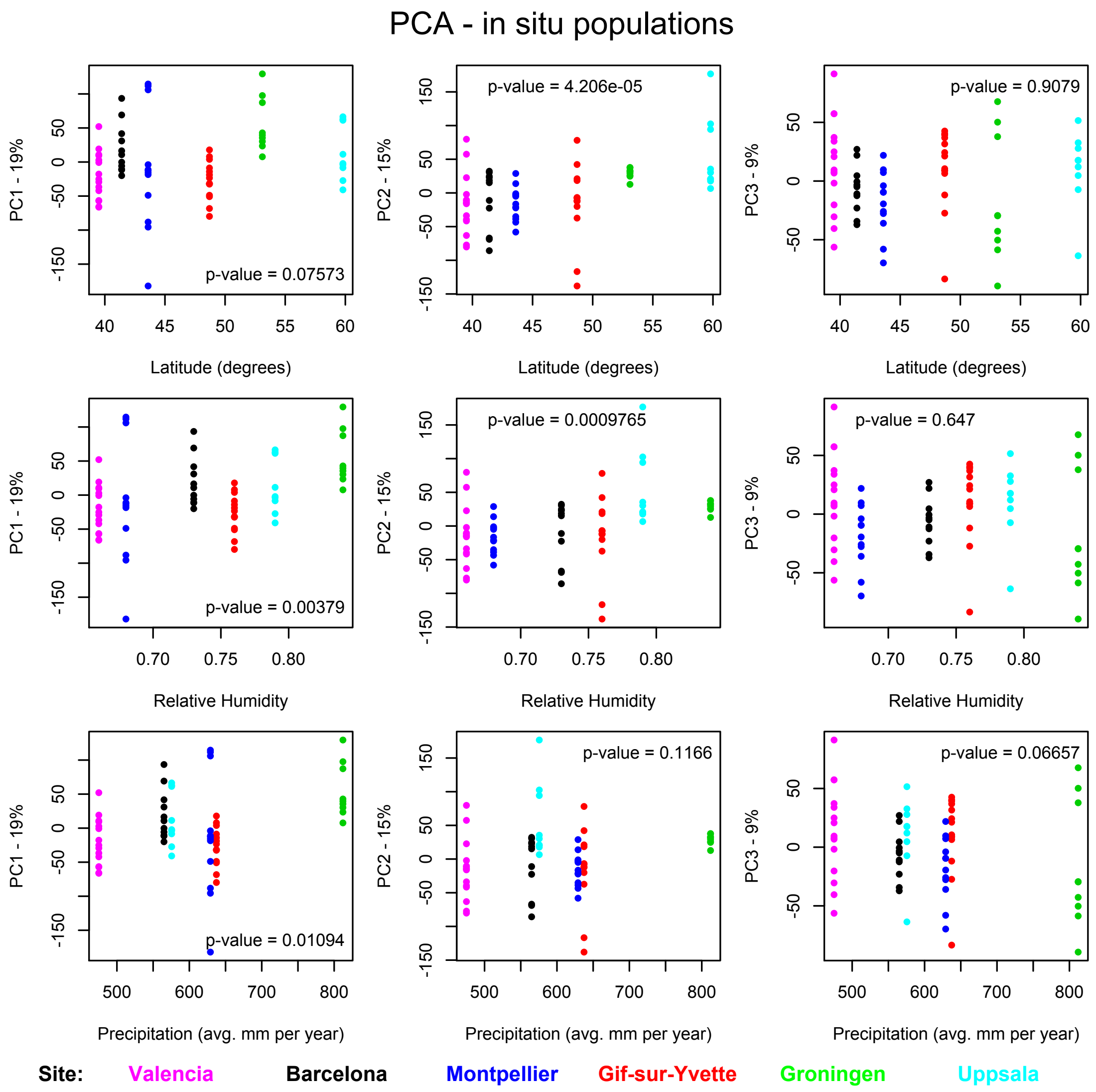

Supplementary Figure 3. **Association between principal components (PC) of in situ populations *RNA-seq* data and abiotic variables**. The three principal components from the PCA analysis on in situ populations *RNA-seq* data were modelled with latitude, which also serves as a proxy for yearly mean temperature (these two have a Pearson's r of -0.9989, see first row of plots), yearly mean relative humidity (second row of plots) and precipitation (third row of plots).

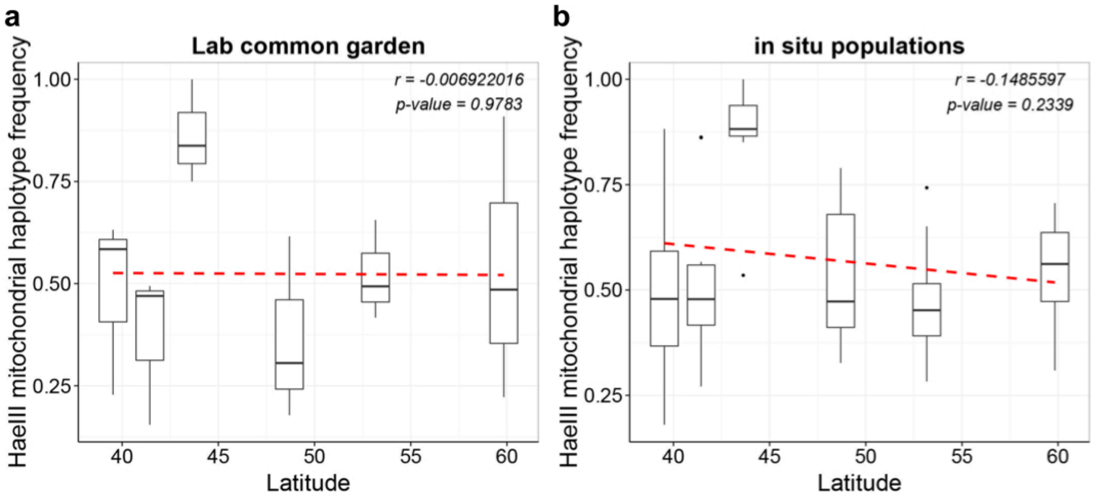

Supplementary Figure 4. ***D. subobscura* populations show no apparent clinal variation in major mitochondrial DNA haplotypes**. Frequencies of the “A” allele at HaeIII restriction site within the *ND5* gene are modelled with latitude to estimate clinal variation of the two major mitochondrial DNA haplotypes in *D. subobscura* (see Methods).

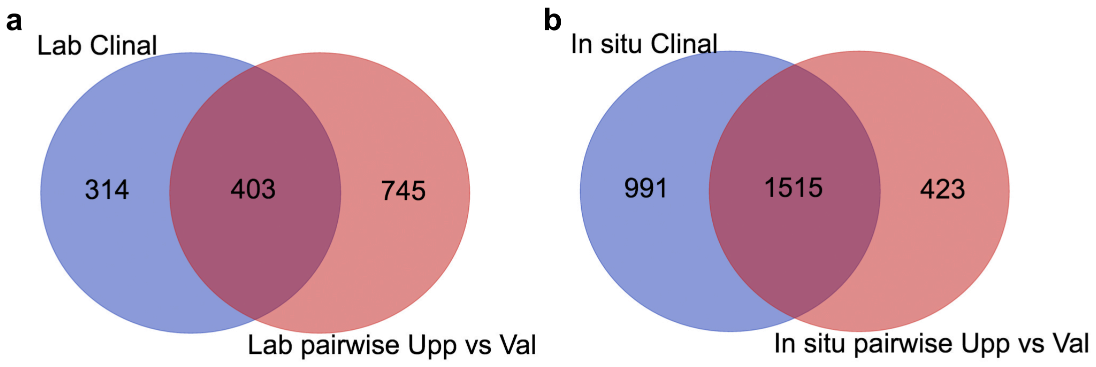

Supplementary Figure 5. **Pearson correlation (clinal) analysis versus pairwise comparison**. The overlaps of significant (1%FDR) genes between the Pearson correlation analysis (clinal) and the pairwise comparison (Uppsala vs Valencia) for the lab common garden (a) and the *in situ* (b) data sets are shown.

Supplementary Tables:

Supplementary Table 1. Drosophila subobscura populations and climatic data associated with their location of origin.

Site	Latitude	Longitude	Population collection	Temperature*	Humidity*	Precipitation (mm)*
Uppsala	59.837593	17.625477	August 2011	6.35	0.79	575.6
Groningen	53.167225	6.546316	August 2011	9.65	0.84	812.1
Gif-sur-Yvette	48.695669	2.076141	August 2011	12.1	0.76	637.4
Montpellier	43.631584	3.815985	September 2011	15.3	0.68	629.1
Barcelona	41.441956	2.104852	September 2011	16.35	0.73	640
Valencia	39.527108	-0.499663	September 2011	17.65	0.66	475

*yearly mean

Supplementary Table 2. Adaptive clinal expression is not an artefact of variation in genes linked to mitochondria, circadian rhythm or thermal stress.

	Functional classes			
	Mitochondrion N=492	Circadian rhythm N=109	Heat stress N=377	Cold stress N=44
Lab common garden CE (all), N=717	34 (P = 0.28)	1 (P = 0.99)	20 (P = 0.76)	2 (P = 0.75)
Lab common garden +CE, N=368	22 (P = 0.09)	1 (P = 0.97)	18 (P = 0.054)	0 (P = 1)
Lab common garden -CE, N=349	12 (P = 0.76)	0 (P 1)	2 (P = 0.99)	2 (P = 0.36)
in situ CE (all) , N=2506	100 (P = 0.53)	19 (P = 0.80)	70 (P = 0.98)	7 (P = 0.91)
in situ +CE, N=1171	62 (P = 0.11)	6 (P = 0.96)	49 (P = 0.09)	5 (P = 0.52)
in situ -CE, N=1335	38 (P = 0.99)	13 (P = 0.59)	21 (P = 1)	2 (P = 0.98)

P (p-value) from Fisher's exact test

Supplementary Table 3. Nonlinear regression modelling of clinal expression patterns.

	**sigmoid**	**linear**	**step (all)**	step 40.5ºN	step 42.5ºN	step 46ºN	step 50ºN	step 56ºN
Lab common garden								
+CE contigs	15	253	100	0	4	54	11	31
-CE contigs	18	252	79	0	6	53	13	7
SUM	33	505	179	0	10	107	24	38
in situ								
+CE contigs	180	412	579	0	7	51	387	134
-CE contigs	498	547	290	120	19	24	111	16
SUM	678	959	869	120	26	75	498	150
